# Post-inflammatory Hyperpigmentation in Skin of Color: Emerging Therapies and Treatment Algorithms

**DOI:** 10.7759/cureus.107234

**Published:** 2026-04-17

**Authors:** Calista Persson, Roma Desai, Christo Manikkuttiyil, Harleen Multani, Raven Lirio, Mikaela Nueva, Roksana Hesari, Akhil Gupta

**Affiliations:** 1 Osteopathic Medical School, Nova Southeastern University Dr. Kiran C. Patel College of Osteopathic Medicine, Fort Lauderdale, USA; 2 Medicine, University of Kentucky College of Medicine, Lexington, USA; 3 Osteopathic Medicine, Nova Southeastern University Dr. Kiran C. Patel College of Osteopathic Medicine, Fort Lauderdale, USA; 4 Medicine, Meharry Medical College, Nashville, USA; 5 Medicine, A.T. Still University - Kirksville College of Osteopathic Medicine, Kirksville, USA; 6 Medicine, Touro College of Osteopathic Medicine, Harlem, USA; 7 Dermatology, Larkin Community Hospital Palm Springs Campus, Hialeah, USA; 8 Dermatology, Skin Center of Florida, North Palm Beach, USA

**Keywords:** chemical peels, cysteamine, hyperpigmentation treatment, laser therapy, pediatric dermatology, post-inflammatory hyperpigmentation, skin of color, tranexamic acid

## Abstract

Post-inflammatory hyperpigmentation (PIH) is a common pigmentary disorder that is especially prevalent among individuals with skin of color. Lesions can persist for months to years and may contribute to psychosocial distress. Given its chronicity and significant impact, it is important to optimize treatment approaches tailored to diverse skin types. This review evaluated emerging therapies and treatment algorithms for PIH across adult and pediatric populations, with a focus on evidence specific to skin of color. A comprehensive literature search covering 2015 to 2025 identified clinical studies including randomized controlled trials (RCTs), cohort studies, case series, and case reports that reported on topical, systemic, or procedural interventions for PIH. Emphasis was placed on therapies that were evaluated in skin-of-color cohorts.

Topical agents such as hydroquinone (HQ), retinoids, azelaic acid (AA), cysteamine, and tranexamic acid (TXA) remain first-line therapies alongside photoprotection. Recent clinical trials have demonstrated their efficacy: cysteamine 5% cream reduced hyperpigmentation indices within 16 weeks, while topical TXA combinations yielded a 30-40% improvement in pigmentation with favorable safety. Oral TXA and emerging nutraceuticals have also shown promise as systemic options. Procedural interventions, including chemical peels and laser therapies, must be used cautiously in individuals with darker skin. In pediatric PIH, first-line management includes gentle skincare, sun protection, and treatment of the underlying inflammatory condition before considering more aggressive interventions. Emerging therapies such as cysteamine and TXA expand the range of treatment options for PIH, providing effective and safe alternatives to HQ in skin of color. Stepwise, individualized regimens that integrate topical therapy, photoprotection, and adjunctive interventions can optimize outcomes while minimizing risks. Further research in pediatric and skin-of-color populations is needed to refine treatment algorithms.

## Introduction and background

Post-inflammatory hyperpigmentation (PIH) is an acquired hypermelanosis that develops after cutaneous inflammation or injury, presenting as tan, brown, or gray macules and patches at the sites of prior insult [1]. Although PIH can occur in all ethnic groups, it is especially common and often more severe in individuals with skin of color (Fitzpatrick skin phototypes IV to VI), where lesions may persist for months or even years if untreated [1,2]. Common causes include acne, atopic or contact dermatitis, arthropod bites, trauma, and other inflammatory dermatoses [1]. In addition, certain cultural and cosmetic practices in patients with skin of color - such as tight hairstyling, chemical hair treatments, and the use of fragranced or irritant skincare products, as well as unregulated skin-lightening agents - may contribute to inflammation and increase the risk of PIH, particularly when they induce irritant or allergic contact dermatitis [3-5].

**Figure 1:**
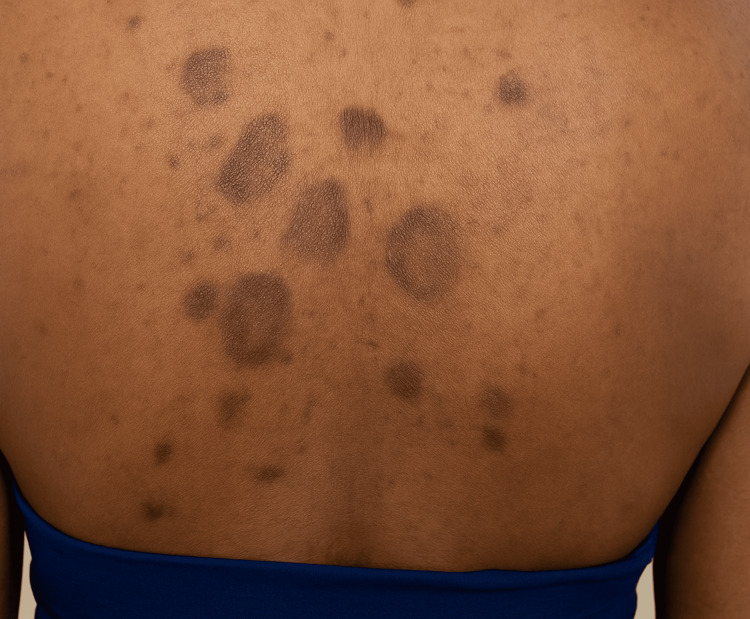
Post-inflammatory hyperpigmentation in skin of color

The psychosocial burden of PIH is considerable; patients frequently report distress and reduced self-esteem, and in some cases, the impact on quality of life may exceed that of the preceding dermatosis [1,2]. Recognition of culturally relevant contributors is important for both prevention and management, as addressing underlying triggers, minimizing irritation, and initiating early, appropriate therapy may improve outcomes and reduce the risk of recurrence [3,5]. Figure 1 depicts representative clinical features of PIH in skin of color, highlighting the contrast of hyperpigmented macules and patches against darker baseline skin tones. This visualization underscores both the visibility and chronicity of PIH, particularly when lesions arise after acne or eczema, and reflects the disproportionate psychosocial burden experienced by patients with richly pigmented skin [1,2].

Management of PIH in richly pigmented skin requires a tailored approach. Because patients with skin of color are more susceptible to treatment-related pigmentary changes, efficacy must be balanced with safety, and irritant or inflammatory side effects should be minimized [3]. Photoprotection is universally recognized as the cornerstone of both prevention and treatment, with daily use of broad-spectrum, high-SPF sunscreens (including agents that protect against visible light) shown to help mitigate darkening and recurrence [2,3].

Conventional topical therapies such as hydroquinone (HQ), retinoids, azelaic acid (AA), and superficial chemical exfoliants remain widely used. HQ has long been considered the gold standard, either alone or as part of triple-combination (TC) therapy; however, prolonged use is limited by the risk of rebound pigmentation and exogenous ochronosis [4]. Retinoids and AA are commonly used adjuncts that accelerate epidermal turnover, reduce melanin, and treat acne, which is one of the most frequent underlying causes of PIH [5]. Despite their effectiveness, these agents require careful monitoring in darker phototypes to avoid worsening due to irritation.

In recent years, newer therapies such as cysteamine, tranexamic acid (TXA), and novel tyrosinase inhibitors (e.g., isobutylamido-thiazolyl-resorcinol) have shown promising results in reducing pigmentation in skin of color, offering safer alternatives or adjuncts to hydroquinone [4,6,7]. Procedural interventions, including chemical peels, microneedling, and laser therapies, have also been explored but must be applied cautiously in darker skin types due to the higher risk of post-treatment hyperpigmentation [8,9].

Despite its prevalence, the evidence base for PIH treatment in skin of color remains limited. Many trials have focused on melasma or other pigmentary disorders and only secondarily reported outcomes in PIH, making extrapolation of findings necessary [2]. Additionally, inflammatory dermatoses in skin of color may present with more brownish hues and are often accompanied by coexisting PIH, further complicating diagnosis and management [5]. Pediatric PIH is particularly under-studied, with recommendations largely derived from adult data [1]. These gaps underscore the need for a more systematic evaluation of both established and emerging therapies tailored to diverse populations.

This review synthesizes clinical studies published between 2015 and 2025 on the management of PIH, emphasizing outcomes in skin of color. We summarize topical, systemic, and procedural therapies, highlight emerging interventions, and propose stepwise treatment algorithms for adults and children to optimize outcomes while minimizing risk.

## Review

Methods

**Figure 2:**
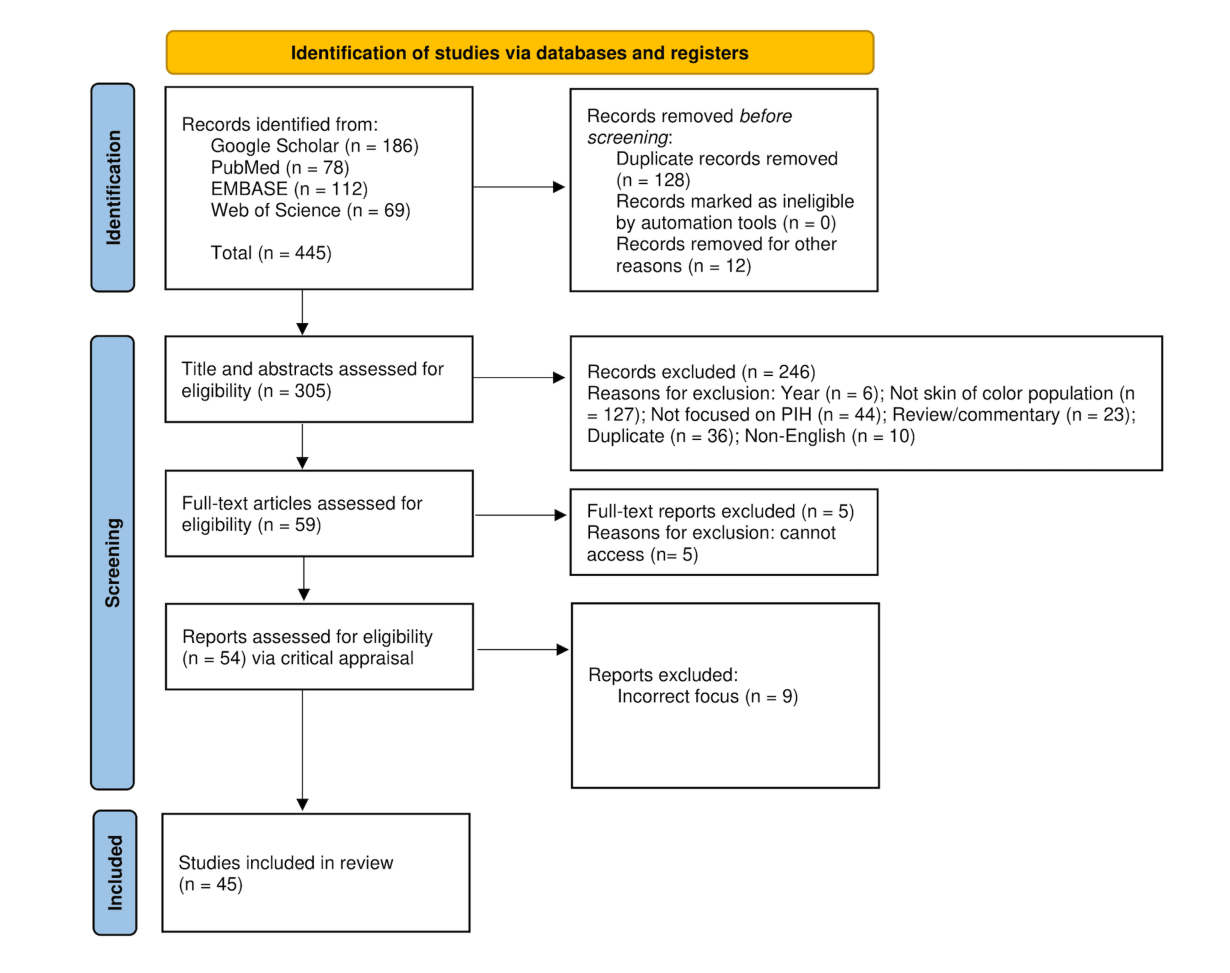
PRISMA flow diagram illustrating the selection of studies* ^*^[10] PRISMA: Preferred Reporting Items for Systematic Reviews and Meta-Analyses

A comprehensive literature search was conducted to identify studies published between 2015 and 2025 evaluating treatments for PIH, with particular emphasis on skin-of-color populations. The databases PubMed, Google Scholar, EMBASE, and Web of Science were systematically searched using combinations of the following terms: “post-inflammatory hyperpigmentation,” “skin of color,” “cysteamine,” “tranexamic acid,” “chemical peel,” “laser,” “pediatric,” and “facial hyperpigmentation.” Manual reference screening of relevant articles was also performed to identify additional studies. The review methodology followed the Preferred Reporting Items for Systematic Reviews and Meta-Analyses (PRISMA) guidelines [10]. The study selection process is illustrated in Figure 2. A total of 445 records were identified across the databases (Google Scholar n = 186, PubMed n = 78, EMBASE n = 112, and Web of Science n = 69). Before screening, 128 duplicate records and 12 records that were removed for other reasons were excluded, leaving 305 records for title and abstract screening. During this stage, 246 records were excluded due to publication years being outside the inclusion range (n = 6), lack of inclusion of skin-of-color populations (n = 127), studies not focused on PIH (n = 44), additional duplicate records (n = 36), and non-English publications (n = 10).

Following initial screening, 59 full-text articles were assessed for eligibility. Five full-text reports were excluded due to the inaccessibility of the full text. The remaining 54 reports underwent critical appraisal, during which nine studies were excluded because they did not primarily focus on PIH treatment outcomes. Ultimately, 45 studies met the inclusion criteria and were included in the final review. Eligible studies included randomized controlled trials (RCTs), nonrandomized clinical trials, cohort studies, case series, case reports, narrative reviews, and systematic reviews that reported on treatment approaches, efficacy outcomes, or safety considerations for PIH. Particular emphasis was placed on novel therapies (e.g., cysteamine, tranexamic acid), laser and light-based devices, chemical peels, and combination regimens, especially in studies including Fitzpatrick skin types IV to VI or explicitly defined skin-of-color populations.

Extracted data included participant demographics, Fitzpatrick skin type, intervention modality, treatment duration, outcome measures, and reported adverse effects. Due to substantial heterogeneity in study design, patient populations, and outcome measures, a quantitative meta-analysis was not feasible. Therefore, findings were synthesized descriptively by treatment modality, with emphasis on higher-level evidence and relevance to skin-of-color populations. All figures and schematic illustrations presented in this article, including the treatment algorithm and PRISMA diagram, were created by the authors using software such as Adobe Acrobat and Microsoft Paint for graphic design and layout.

Results

Across the included studies, acne was the most common cause of PIH, followed by eczematous dermatitis, cosmetic procedures, and trauma or burns. Most investigations concentrated on facial PIH, although several also examined lesions on the trunk and extremities. All studies included patients with skin of color (Fitzpatrick III-VI), representing Asian, African, and Hispanic cohorts and highlighting the global burden of PIH in darker skin tones [2]. Outcome measures varied, encompassing clinical grading scales (e.g., physician’s global assessments, pigmentation severity indices), adapted tools such as the Melasma Area and Severity Index (MASI) and the Post-Acne Hyperpigmentation Index, objective instruments (melanin index, spectrophotometry), and patient-reported outcomes like satisfaction and quality-of-life assessments [10].

Interventions were broadly grouped into topical, systemic, and procedural therapies. The following sections summarize results for each category, with emphasis on emerging options and their role alongside established treatments.

Topical Therapies

HQ and triple combination cream: HQ remains the benchmark depigmenting agent. Applied once or twice daily in 2-4% formulations, it can visibly lighten PIH over 12-16 weeks. In a Nigerian cohort of post-acne PIH patients, 4% HQ used for eight weeks produced a mean melanin index reduction of ~25% compared with baseline [11]. The most robust results are achieved with the classic TC formula-HQ, tretinoin, and a corticosteroid, which is considered the gold standard for recalcitrant hyperpigmentation. In an Indian RCT of 60 patients, a modified Kligman’s formula (HQ 4%, tretinoin 0.05%, mometasone furoate 0.1%) achieved a ~44% pigment reduction after 12 weeks, compared with only 8% in the placebo group [8]. Importantly, no rebound hyperpigmentation or ochronosis was reported in this short-term study.

Nevertheless, HQ use is tempered by safety concerns. Irritant or allergic contact dermatitis can exacerbate PIH, and long-term or unsupervised application beyond four to six months has been associated with exogenous ochronosis, a paradoxical bluish-black pigmentation [12]. These risks have led to restrictions on HQ availability in some regions and underscore the need for alternative or maintenance therapies.

Conventional non-HQ topicals: Topical retinoids (tretinoin, adapalene, tazarotene) promote keratinocyte turnover, disperse melanin granules, and reduce inflammation from acne and dermatitis. While initial irritation may transiently darken PIH in sensitive skin, gradual introduction of moisturizers usually mitigates this. Clinical trials demonstrate benefit: tretinoin 0.1% and adapalene 0.1% gels applied for 12 weeks led to partial clearance of post-acne PIH in diverse skin-of-color cohorts [2,5]. In African American patients, nightly tretinoin 0.1% for six months resulted in good-to-excellent improvement in 50% of participants [13]. Tazarotene 0.1% has also been effective when used long-term [5].

AA is another safe and effective option. At 20% concentration, it competitively inhibits tyrosinase and reduces oxidative stress. A randomized trial showed comparable outcomes between AA 20% and HQ 4%, with ≥50% pigment reduction achieved in 62% of patients in the AA arm and fewer irritant reactions [14]. Because of its tolerability, AA is often used for maintenance, especially in acne-prone or rosacea-prone patients. Niacinamide (vitamin B3) and vitamin C (ascorbic acid) are widely used adjuncts. Niacinamide 5% reduced melanosome transfer and produced moderate lightening over 12 weeks in Brazilian patients, though results plateaued without additional therapy [15]. Stabilized vitamin C (10-20%) reduces oxidized melanin and enhances other agents; a 15% ascorbic acid serum achieved mild-to-moderate improvement over 12 weeks in Filipino patients [16]. Overall, these non-HQ topicals are modestly effective but safe, making them useful as adjuncts or maintenance options [1].

**Figure 3:**
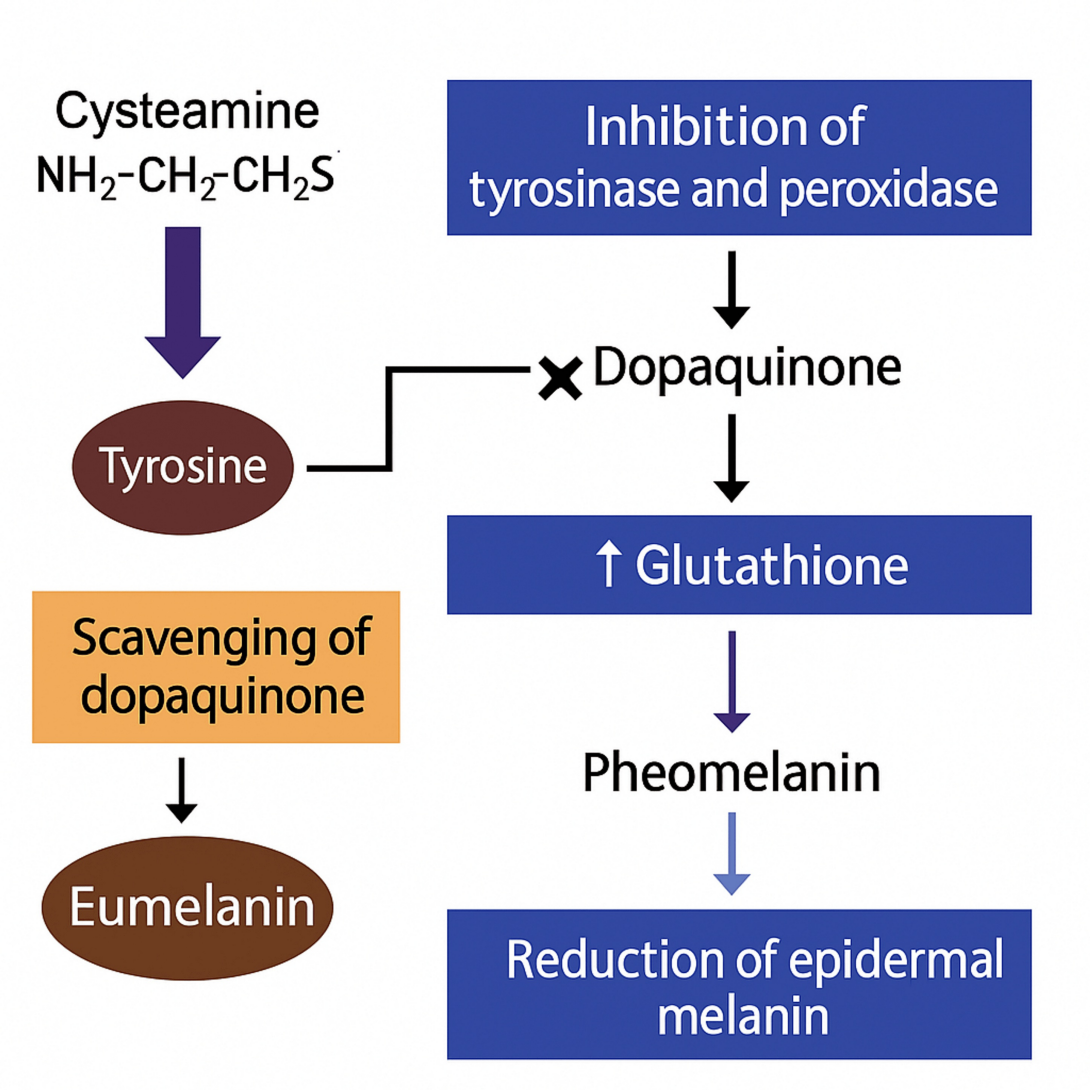
Mechanism of cysteamine in inhibiting melanogenesis Image credit: authors

Cysteamine cream: Cysteamine is a cysteine-derived molecule that targets multiple steps in melanogenesis (Figure 3). It inhibits tyrosinase and peroxidase enzymes, blocking conversion of tyrosine to dopaquinone, the rate-limiting step in melanin synthesis [17]. It scavenges dopaquinone, preventing its polymerization into melanin polymers, and increases intracellular glutathione, shifting synthesis toward lighter pheomelanin [18]. Cysteamine may also reduce existing epidermal melanin. Collectively, these multimodal mechanisms lighten hyperpigmentation while sparing melanocytes, reducing the risk of cytotoxic damage observed with HQ [11]. This multimodal action likely contributes to its success in complex hyperpigmentation cases. 

Several studies demonstrate cysteamine’s efficacy in PIH and related disorders. In a double-blind randomized trial, 40 patients with Fitzpatrick III-V skin and post-acne or post-inflammatory lesions were treated with a cysteamine-isobionicamide complex once daily for 16 weeks. The treatment arm achieved ~45% improvement in pigmentation indices compared with ~20% in the vehicle group, and colorimetric melanin index scores were significantly reduced [19]. Optical coherence tomography confirmed decreased melanosome “capping” in basal keratinocytes, suggesting a structural reduction in pigment. Patients also reported improved quality-of-life scores.

Case reports further support its use in refractory PIH. For example, a patient unresponsive to eight months of TC cream experienced marked clearance of residual lesions after 12 weeks of nightly cysteamine 5% [20]. Safety across studies has been favorable, with only transient redness or tingling reported. Importantly, cysteamine has not been associated with ochronosis, even with extended use [21,22]. Limitations include a sulfur-like odor and application requirements that involve short contact times followed by wash-off [23]. Despite these drawbacks, cysteamine is increasingly recognized as a viable first-line or maintenance alternative to HQ in skin of color.

**Figure 4:**
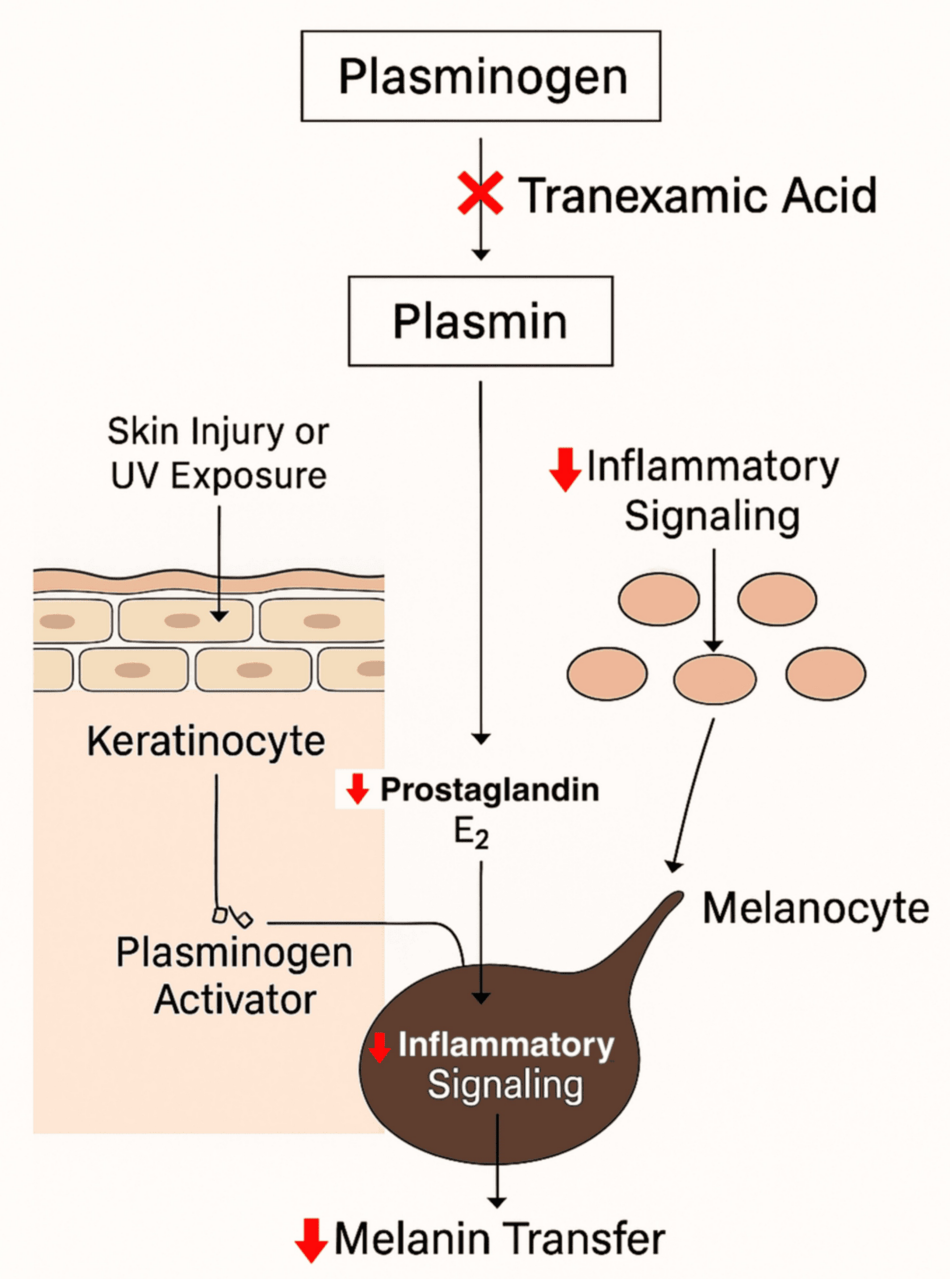
Mechanism of tranexamic acid in post‑inflammatory hyperpigmentation Image credit: authors

Topical TXA: TXA is a synthetic lysine analog that targets the plasminogen-plasmin pathway (Figure 4). Following skin injury or ultraviolet exposure, keratinocytes release plasminogen activator, leading to plasmin formation. Plasmin stimulates inflammatory mediators such as prostaglandin E2 and α-MSH, which upregulate melanogenesis and promote melanin transfer to keratinocytes [24]. By blocking the conversion of plasminogen to plasmin, TXA reduces this inflammatory signaling. It also exerts direct anti-inflammatory effects, thereby interrupting keratinocyte-melanocyte crosstalk and decreasing pigment production [25].

TXA has been studied in both topical and systemic formulations for PIH, often in combination with other agents. In an open-label trial of 50 women with mixed hyperpigmentation, a multimodal cream containing TXA, niacinamide, and licorice extract produced a 37% reduction in pigment intensity and a 45% improvement in skin brightness after 16 weeks, with excellent tolerability and no irritant reactions [26]. Topical TXA at concentrations of 2-5% has shown moderate but consistent efficacy in smaller studies of PIH and melasma, with enhanced outcomes when combined with adjuncts such as niacinamide or licorice [7,27].

Laser-assisted drug delivery has been explored to improve TXA penetration. In a split-face, double-blind randomized trial of post-acne PIH, patients underwent six sessions of picosecond fractional laser over 12 weeks, with TXA applied to one side and a placebo to the other. Blinded assessments showed significantly greater lightening on the TXA-treated side. The authors attributed this to the microchannels created by the fractional laser, which enhanced trans-epidermal delivery of TXA [28]. Unlike vascular-targeting lasers that reduce erythema, this approach was designed specifically to optimize pigment clearance.

Standalone topical TXA is generally well tolerated, with a safety profile superior to hydroquinone or retinoids, making it particularly suitable for sensitive skin. Its effects are most pronounced when used as part of combination regimens or paired with procedural adjuncts. Clinically, TXA provides gradual lightening of existing PIH and helps prevent new lesion development in inflammation-prone skin, making it especially valuable in skin-of-color populations.

Other novel topicals: Several novel and botanical-derived agents have been studied. Isobutylamido thiazolyl resorcinol, a patented tyrosinase inhibitor, significantly reduced hyperpigmented spots in phototypes IV-VI with efficacy comparable to 2% HQ and no reported irritation [4,29]. Other agents, including kojic acid and licorice extract (glabridin), have modest lightening effects and are often incorporated into cosmetic formulations. While safe in skin of color, their impact on PIH is mild as monotherapy, making them more suitable as adjuncts to maintain results after primary therapy [30,31].

Systemic and Intradermal Therapies

Although topical treatments remain the first-line approach for PIH, systemic agents are increasingly considered for patients with diffuse, extensive, or refractory disease. Options include oral retinoids, glutathione, antioxidants, polypodium leucotomos extract, melatonin, and vitamins, most of which are supported by limited or anecdotal evidence [32]. Among these, oral tranexamic acid is the most studied and clinically relevant systemic option.

Oral TXA: TXA was originally developed as a hemostatic agent but was observed to lighten pigmentation in patients with melasma [3]. Although no large randomized trials have evaluated its use in PIH specifically, evidence from melasma has been extrapolated to PIH management. TXA reduces inflammation-driven melanogenesis by inhibiting the plasminogen-plasmin pathway. Several meta-analyses confirm that oral TXA, administered at 500-750 mg/day for 8-12 weeks, significantly reduces pigmentation severity, typically assessed with MASI, with studies demonstrating mean MASI reductions of approximately 30-50% from baseline following treatment [33].

Clinically, oral TXA has been used in PIH for patients with widespread hyperpigmentation, especially in cases where PIH was secondary to aesthetic procedures or severe inflammatory acne. Reviews indicate that it has a favorable safety and efficacy profile compared to other systemic options [34]. Short-term prophylactic use before cosmetic procedures, such as chemical peels or lasers, has also been explored, with some small studies suggesting reduced risk of post-procedure PIH in patients with skin of color, though results are inconsistent and formal guidelines are lacking [2].

Oral TXA is commonly prescribed off-label to adults at 250 mg twice daily. Side effects are generally mild and include gastrointestinal upset or menstrual irregularities. Thromboembolic events have not been reported at dermatologic doses in healthy patients, but careful screening for thrombotic risk factors is essential [35]. Treatment is typically limited to three to six months, after which maintenance therapy is continued with topical agents [7]. Because TXA does not address the underlying inflammatory trigger, recurrence of PIH may occur if conditions such as acne or eczema remain uncontrolled, highlighting the importance of concurrent management of the primary disease. Oral TXA should therefore not be considered routine therapy for all PIH. Its use is best reserved for adults with diffuse or refractory disease, or for short-term prophylaxis in high-risk procedural contexts, and it should be avoided in children and in patients with thromboembolic risk.

Other systemic agents: Other systemic treatments are supported by weaker evidence. Glutathione, frequently marketed as a “master antioxidant,” has been evaluated in oral and intravenous formulations for pigmentary disorders. Oral glutathione (500 mg daily) produced a modest, reversible decrease in UV-induced pigmentation in one randomized placebo-controlled trial, although the clinical relevance for PIH remains uncertain [36]. Glutathione may reduce melanogenesis through antioxidant activity, tyrosinase inhibition, and a shift from eumelanin toward pheomelanin production, but clinical studies evaluating its use for hyperpigmentation remain limited and heterogeneous [12].

N-acetylcysteine (NAC), a precursor to glutathione, has also been proposed as an adjunctive therapy due to its antioxidant properties and theoretical ability to reduce melanocyte activation through oxidative stress modulation. However, clinical evidence supporting NAC for pigmentary disorders is sparse, and robust data supporting its routine use in PIH are lacking [16].

Polypodium leucotomos, a fern extract with antioxidant and photoprotective effects, has demonstrated benefit in melasma and may serve as a useful adjunct in PIH, particularly for patients with significant sun exposure [37]. Oral retinoids, including isotretinoin and acitretin, are occasionally cited for pigmentary benefit, although their primary role is in treating underlying inflammatory conditions such as acne and psoriasis. Patients receiving isotretinoin often report gradual fading of PIH, likely related to improved inflammation control and accelerated epidermal turnover [13]. Other integrative agents, including pycnogenol, licorice root extract, vitamin C, and similar nutraceuticals, have been evaluated in small studies but appear to provide only modest adjunctive benefits [38,39]. While their favorable safety profiles support use as supplemental therapy, they should not replace established evidence-based treatments.

Chemical peels: Chemical peels, when used conservatively, are among the safest procedural options for PIH in skin of color. They exfoliate the upper epidermis, promoting renewal of epidermal cells, leading to more evenly pigmented skin and stimulating dermal remodeling. The most studied α-hydroxy acid, glycolic acid, has shown significant lightening of PIH when used at concentrations of 20-50% every two to four weeks. In an Indian randomized trial, patients treated with serial glycolic acid peels plus topical therapy achieved greater pigment reduction than those on topicals alone, with over half attaining at least 50% clearance after 12 weeks [8]. Salicylic acid peels, which combine keratolytic and anti-inflammatory properties, are effective in acne-prone or oily-skinned patients and have produced results comparable to glycolic acid [7]. Lactic acid peels provide a gentler option, while daily use of ammonium lactate 12% lotion has been reported to gradually fade body PIH in pediatric or sensitive cases [1]. However, stronger peels, such as trichloroacetic acid above 20% or phenol, are not recommended in darker phototypes due to risks of scarring and permanent dyschromia [40].

Laser and light therapies: Lasers and light-based devices can directly target melanin or remodel the dermis, but their application in skin of color requires strict caution due to the increased risk of inducing or worsening PIH. Patients with darker phototypes are also more prone to Koebnerization, a phenomenon in which new lesions or pigmentary changes develop at sites of cutaneous trauma or inflammation, thereby increasing the risk of procedure-induced dyspigmentation in susceptible individuals [6]. Low-fluence Q-switched Nd:YAG 1064 nm laser is the most studied modality and can gradually lighten diffuse post-acne PIH over 6-10 weekly sessions, although rebound hyperpigmentation is observed in a subset of patients after discontinuation [9,41]. Fractional non-ablative lasers, such as the 1927 nm thulium, create microscopic zones of controlled injury that stimulate regeneration and have shown success in recalcitrant cases of PIH, including clearance of facial PIH in Fitzpatrick V skin without scarring [42].

Ablative protocols, including fractional CO₂, remain high-risk, though limited studies suggest potential textural benefits when carefully performed [43]. Ruby and alexandrite lasers (694 and 755 nm) should be avoided in skin of color due to their high melanin absorption and associated risk of burns and dyspigmentation [44,45]. Pulsed-dye lasers, while not pigment-specific, may improve acne-related PIH by reducing vascular erythema that often accompanies hyperpigmentation [46]. Intense pulsed light (IPL), however, is generally contraindicated in darker phototypes because of its non-specificity and high incidence of post-treatment pigmentation [47]. Regardless of modality, strict peri- and post-procedure photoprotection and short-term anti-inflammatory prophylaxis are essential to minimize adverse events [2].

Microneedling and adjunctive therapies: Microneedling provides a lower-risk, heat-free alternative to lasers for PIH in skin of color. By producing controlled micro-injuries, microneedling stimulates epidermal turnover and collagen remodeling, leading to gradual pigment clearance and improvement in texture. Studies have demonstrated meaningful improvements in PIH and acne scars among patients with darker phototypes after serial sessions [48,49]. This procedure is particularly promising in SOC because it avoids the thermal injury associated with lasers, which reduces the risk of paradoxical pigmentation. Microneedling can also be combined with topical depigmenting agents such as vitamin C or TXA to enhance dermal delivery, often producing superior outcomes compared to either modality alone [12]. Platelet-rich plasma, though primarily studied in melasma, has been reported to accelerate clearance when combined with microneedling or laser treatments, likely through the release of growth factors that influence melanocyte activity [50]. Collectively, physical and procedural therapies provide valuable adjunctive options for PIH management in skin of color when used conservatively and in conjunction with topical or systemic agents.

Summary of Treatments for PIH

Overall, treatment of PIH in skin of color relies on a multimodal strategy balancing efficacy and safety. Topical agents remain foundational. HQ and TC regimens produce the most rapid short-term clearance, while retinoids, AA, niacinamide, and vitamin C provide incremental improvement with favorable safety profiles. Cysteamine and topical TXA serve as effective HQ-sparing alternatives. Procedural adjuncts, including chemical peels and fractional lasers, can accelerate clearance when used conservatively. Among systemic options, oral TXA is the only therapy with consistent supporting evidence, demonstrating benefit for diffuse, refractory, or peri-procedural PIH. Its use should be limited to carefully selected adults without thromboembolic risk. Other oral agents, including glutathione, polypodium leucotomos, retinoids, and antioxidants, appear to play only modest or supportive roles. Table 1 summarizes topical, systemic, and procedural therapies for PIH in skin of color, including mechanisms, efficacy, and key considerations.

**Table 1:** Comparison of therapies for PIH in SOC: mechanisms, efficacy, and considerations PIH: post-inflammatory hyperpigmentation; SOC: skin of color; HQ: hydroquinone; AA: azelaic acid; RCT: randomized controlled trial; TXA: tranexamic acid

Therapy	Mechanism of action	Clinical efficacy in PIH	Limitations/considerations
HQ (2-4%)	Inhibits tyrosinase; causes selective melanocyte damage; suppresses melanosome formation [51]	Gold-standard agent; ~30-50% lightening within 8-12 weeks [11]; enhanced efficacy in triple-combination regimens [8]	Irritation is common; prolonged continuous use is discouraged due to ochronosis risk [12]; misuse may cause rebound PIH; regulated in some regions
Retinoids (tretinoin, adapalene, tazarotene)	Increase keratinocyte turnover; disperse melanin; anti-inflammatory [5]	Partial clearance (~30-40%) after 3-6 months [2]; 50% achieved good-to-excellent improvement with tretinoin 0.1% in African American patients [13]	Initial irritation may transiently worsen PIH; requires sunscreen and moisturizer; best used in combination
AA 20%	Reversible tyrosinase and oxidoreductase inhibitor; anti-inflammatory	~25-50% improvement over 3-4 months; efficacy comparable to HQ 4% with fewer adverse effects [14,52]	Mild tingling or dryness possible; safe for long-term use and pregnancy; useful for maintenance [8]
Niacinamide (4-5%)	Reduces melanosome transfer; barrier stabilizer; anti-inflammatory	Mild-to-moderate improvement (~20-35% over 8-12 weeks) [15]; enhances results when combined with TXA or vitamin C [53]	Very well tolerated but modest efficacy as monotherapy; often used in combination regimens [26]
Cysteamine 5%	Inhibits tyrosinase and peroxidases; scavenges dopaquinone; increases glutathione and shifts melanogenesis toward pheomelanin [11]	RCT: ~45% pigmentation reduction over 16 weeks vs. ~20% vehicle [19]; case reports show efficacy in HQ-resistant PIH [20]; effective in melasma [54]	Sulfur-like odor; requires timed application and wash-off [23]; mild irritation possible; no ochronosis reported [21,22]
TXA (topical)	Inhibits plasminogen activation, reducing prostaglandins and α-MSH signaling [24]	TXA + niacinamide cream achieved ~37% pigment reduction over 16 weeks [26]; laser-assisted TXA improved clearance in split-face trials [28]	Excellent tolerability; less potent than HQ alone; best used in combination; requires ≥3 months of use
Oral TXA	Systemic plasmin inhibition reduces melanocyte stimulation and UV-induced vascular mediators	~30-50% improvement with 250 mg twice daily for 3-6 months [33,55]; beneficial for diffuse PIH and peri-procedural prophylaxis [2]	Off-label; contraindicated in patients with thromboembolic risk; recurrence may occur after discontinuation [32,35]
Chemical peels (glycolic, salicylic, lactic acids)	Remove epidermal pigment and stimulate renewal and collagen remodeling	GA peels (20-50%) every 2-4 weeks improve PIH within 2-4 sessions; GA + HQ cleared lesions faster than HQ alone [8]; SA peels are effective for post-acne PIH	Risk of irritation or worsening PIH if overly aggressive; must be conservative in SOC; strict photoprotection required; operator-dependent [1,40]
Lasers and light devices	Target melanin or dermal pigment (QS Nd:YAG, picosecond, fractional); some target vascular components	Low-fluence QS 1064-nm improves diffuse PIH over 5-10 sessions [41]; 1927-nm fractional lasers are effective for resistant cases [42]	High rebound PIH risk if aggressive; conservative settings and prophylaxis required; 10-36% post-laser PIH risk in Asian cohorts [2,9,32,56]

Discussion

Clinical Practice Implications

Management of PIH in skin of color is complex but increasingly achievable with the combined use of established and emerging therapies. Topical treatments remain the cornerstone of management, consistent with expert consensus [3]. HQ continues to be the most effective depigmenting agent when used in short, supervised courses. Trials confirm that HQ 4% alone or in TC formulations can produce significant improvement in as little as eight weeks [8]. However, concerns about rebound pigmentation, exogenous ochronosis, and regulatory restrictions limit long-term or unsupervised HQ use, and many patients prefer alternatives.

Emerging topical therapies such as cysteamine and TXA offer promising options with efficacy comparable to HQ but without its long-term risks. Cysteamine 5% has been validated in RCTs and case series to significantly reduce pigmentation with only minimal transient irritation, and it can be used as a monotherapy or rotated with HQ for maintenance [6,11,19,20]. TXA is similarly versatile: topical formulations reduce pigment and inflammation, making them particularly useful in acne-associated PIH [26,27], while oral TXA, though off-label, has shown benefit in diffuse or refractory cases and as peri-procedural prophylaxis, provided careful patient selection and monitoring are in place [2,33,55].

Importance of Combination Therapy

Evidence strongly supports the use of multi-agent regimens in PIH. Combining a tyrosinase inhibitor such as HQ or cysteamine with a keratolytic or retinoid and an anti-inflammatory agent enhances clearance while reducing irritation. This principle mirrors the original Kligman’s formula, which combined agents with complementary mechanisms. Recent work shows that multi-ingredient topical formulations, such as those containing TXA and niacinamide, can achieve more consistent improvement with fewer side effects than high-strength monotherapy [26,51]. For patients with skin of color, this synergy is particularly important, as aggressive monotherapies can provoke irritation and paradoxical darkening.

Role of Procedural Therapies

Our findings reinforce a cautious but not exclusionary stance on procedural therapies in skin of color. Chemical peels, particularly glycolic acid at 30-50%, are widely used worldwide and have consistently demonstrated efficacy when performed at superficial depth with careful pre- and post-care. Complication rates remain low when patients are appropriately selected and adherent to photoprotection [1,8].

Lasers present greater challenges due to their higher risk profile, but technological advances such as picosecond devices and fractional non-ablative systems have expanded safe options for darker phototypes. Reviews suggest that longer-wavelength devices like low-fluence Q-switched 1064 nm Nd:YAG and fractional 1927 nm thulium lasers can provide incremental improvement when conservative parameters are used [9,42]. Success is greatest when lasers are combined with topical therapy or systemic TXA, which may mitigate rebound pigmentation [57,58]. Adjunctive prophylaxis with short-term topical corticosteroids or calcineurin inhibitors and strict sunscreen use is also increasingly recommended [2,32].

Pediatric and Extrafacial PIH

High-quality evidence for pediatric PIH is lacking. Current management is largely adapted from adult regimens, though a conservative approach is emphasized. The priority is treatment of the underlying inflammatory dermatosis to prevent ongoing pigmentation. In most children, PIH will fade gradually over months to years once triggers are controlled. Gentle skin care, avoidance of irritants, and daily sunscreen are critical, even in childhood [1,59]. For adolescents, agents such as AA or adapalene are considered safe and effective, while stronger agents like HQ or peels are reserved for selected cases with close monitoring [15,60]. Ammonium lactate lotions may help lighten body PIH during a “watchful waiting” period [1].

Extracutaneous sites such as the trunk and limbs present additional challenges. Thicker epidermis can require stronger or more prolonged treatment, yet tolerance may also be greater. Body PIH is commonly triggered by arthropod bites, scratches, or eczema, and often resolves more slowly than facial lesions [51]. Topical regimens combining retinoids at night with HQ or cysteamine in the morning are frequently used, although improvement may take six months or longer [5,6,13]. Lasers such as QS Nd:YAG have been reported for body PIH, but outcomes can be inconsistent and carry a risk of mottled pigmentation [61]. Patient counseling, including avoidance of scratching and adherence to sunscreen, remains essential to prevent new lesions.

Proposed Treatment Algorithm

**Figure 5:**
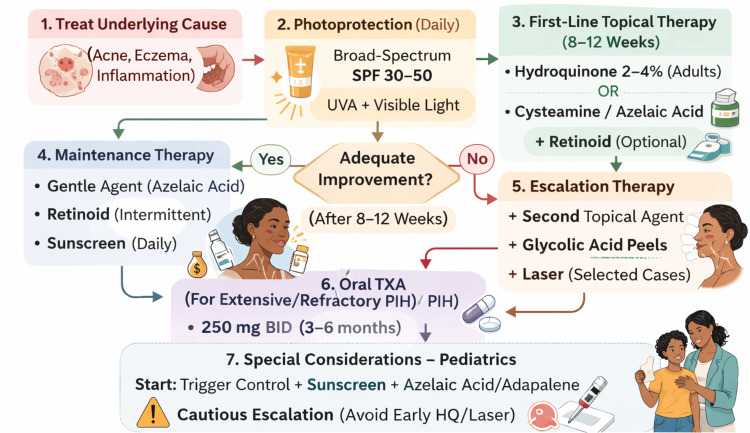
Stepwise treatment algorithm for PIH in skin of color PIH: post-inflammatory hyperpigmentation; HQ: hydroquinone; TXA: tranexamic acid; SPF: Sun Protection Factor; UVA: ultraviolet A radiation; BID: twice daily Image credit: authors

Integrating the available evidence, a stepwise treatment algorithm for PIH in skin of color is proposed (Figure 5). The management begins with the identification and control of the underlying inflammatory condition, such as acne or eczema, to prevent new lesions. At the same time, consistent photoprotection with broad-spectrum sunscreens that block UVA and visible light is essential to prevent progression [62].

First-line therapy consists of a topical depigmenting agent such as HQ 2-4% in adults, or alternatives like cysteamine or AA in those unable to tolerate HQ. Retinoids may be added to enhance epidermal turnover. After 8-12 weeks, clinical response should be reassessed [58]. If significant improvement is achieved, patients can transition to maintenance regimens with gentler agents or continue intermittent use of retinoids and strict sunscreen [13]. If improvement is limited or slower than desired, escalation may include a second topical agent, serial glycolic acid peels, or, in selected cases, laser therapy under conservative protocols [2,6,9]. Oral TXA can be considered for extensive or recalcitrant disease, particularly in peri-procedural settings, provided contraindications are excluded [55].

In pediatric patients, the same principles apply, but escalation is pursued with greater caution. The management typically begins with trigger control, photoprotection, and gentle agents such as AA or adapalene, reserving HQ or procedural interventions for older children or adolescents with significant distress. Throughout care, patient and family education is crucial: expectations should be realistic, adherence emphasized, and the risk of relapse discussed if inflammation recurs [1].

Limitations and future research directions

This review highlights progress in PIH management but also identifies significant gaps. Variability in outcome measures across studies complicates direct comparison, though initiatives to standardize trial endpoints are currently underway [62]. Sample sizes remain small, particularly for emerging therapies such as cysteamine, which limits their generalizability. Long-term follow-up is rare, leaving unanswered questions about durability and potential late safety outcomes. Larger, ethnically diverse, and longer-duration studies are needed.

Future innovations include melanogenesis-targeted molecules such as melanocortin-1 receptor antagonists and Wnt pathway modulators, as well as genetic or siRNA-based approaches that could offer more highly specific interventions [63-65]. Preventive dermatology also deserves attention, including early treatment of acne, peri-procedural prophylaxis with TXA or topical inhibitors, and patient education regarding sun avoidance and photoprotection [2,32].

In summary, PIH management in skin of color requires individualized, stepwise care that integrates photoprotection, topical depigmenting agents, systemic options such as oral TXA, and carefully selected procedures. HQ remains effective but is increasingly supplemented or replaced by alternatives such as cysteamine and TXA. Chemical peels and microneedling are safe and effective adjuncts, while lasers should be reserved for refractory cases in expert hands. Pediatric and extrafacial PIH demand a conservative approach, emphasizing prevention and patience. Looking ahead, standardized outcomes, larger trials, and novel molecular therapies will further refine treatment strategies.

## Conclusions

PIH in skin of color is a prevalent and impactful condition that requires careful, individualized management. Although PIH often resolves slowly and can significantly affect quality of life, recent advances have expanded the therapeutic landscape and created new opportunities for safe and effective treatment. HQ remains an important short-term option, but its potential risks and limitations highlight the value of HQ-sparing alternatives. Among these, cysteamine and TXA have emerged as well-tolerated therapies with growing evidence of efficacy in diverse populations. Traditional agents such as retinoids and AA also remain essential components of therapy, particularly when incorporated into combination regimens that target multiple pathways to enhance pigment clearance while minimizing irritation. Procedural treatments - including chemical peels, microneedling, and carefully selected laser therapies - may provide additional benefits in select patients when performed with conservative parameters and strict photoprotection. Oral TXA represents the most promising systemic therapy but should generally be reserved for adults with diffuse or treatment-resistant PIH after careful risk assessment. Other oral agents, such as glutathione, polypodium leucotomos, and antioxidants, may offer supportive benefit but should not replace established first-line therapies. Overall, management of PIH in skin of color is best approached with a stepwise strategy beginning with photoprotection and topical lightening agents, followed by escalation to systemic or procedural therapies when necessary, while emphasizing patient education, adherence, and realistic expectations. Continued research, particularly larger and longer-term clinical studies, will further refine treatment strategies and improve outcomes for affected patients.
